# Supplementary material for: Biomimetic Bouligand chiral fibers array enables strong and superelastic ceramic aerogels
Source: Nat Commun. 2024 Jan 6;15:336. doi: 10.1038/s41467-023-44657-2 (PMC10771491; doi:10.1038/s41467-023-44657-2)
Supplement: Supplementary file 1 — Supplementary Information [file 41467_2023_44657_MOESM1_ESM.pdf]

Supplementary Information

Biomimetic Bouligand Chiral Fibers Array Enables Strong and Superelastic Ceramic Aerogels

Wang *et al.*

Supplementary Figures

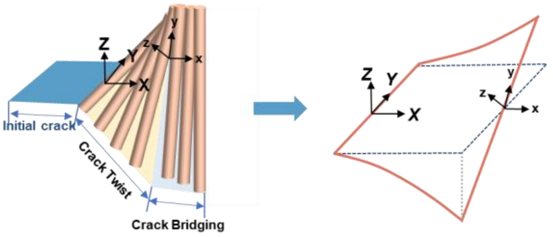

Supplementary Figure 1. Diagram of the mechanical fracture model, which takes into account twist formation.

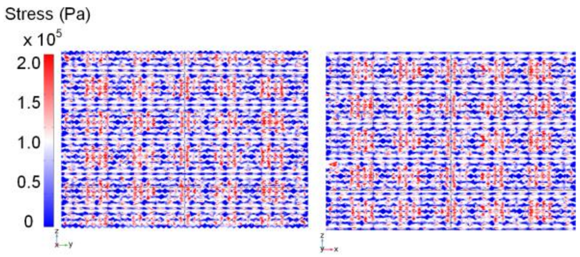

Supplementary Figure 2. Stress nephograms of the Bouligand structure depict the changes in axial stress in the *y-z* (left) and *x-z* (right) planes.

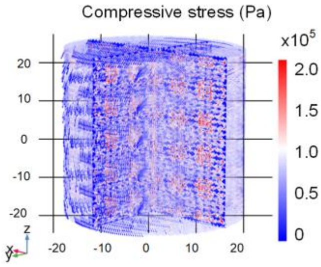

Supplementary Figure 3. In-plane isotropy of the mechanical properties in compression mechanics tension mode.

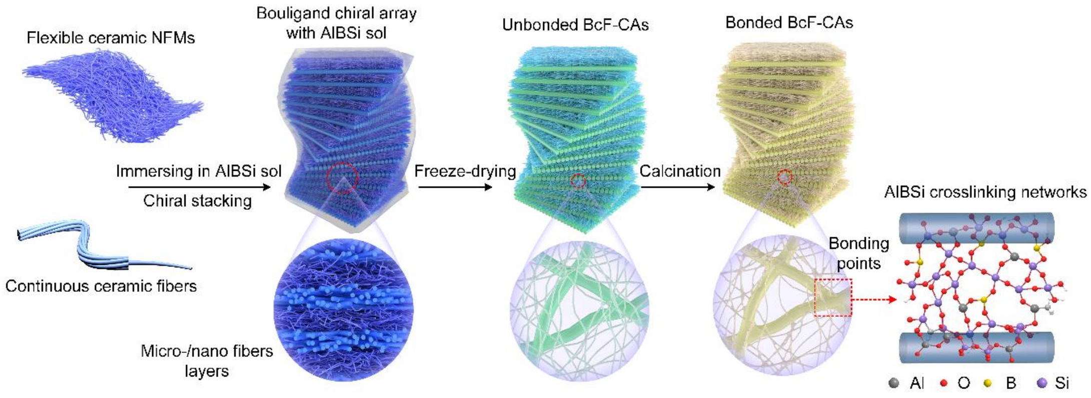

Supplementary Figure 4. Schematic illustration of the manufacturing process of the BcF-CAs.

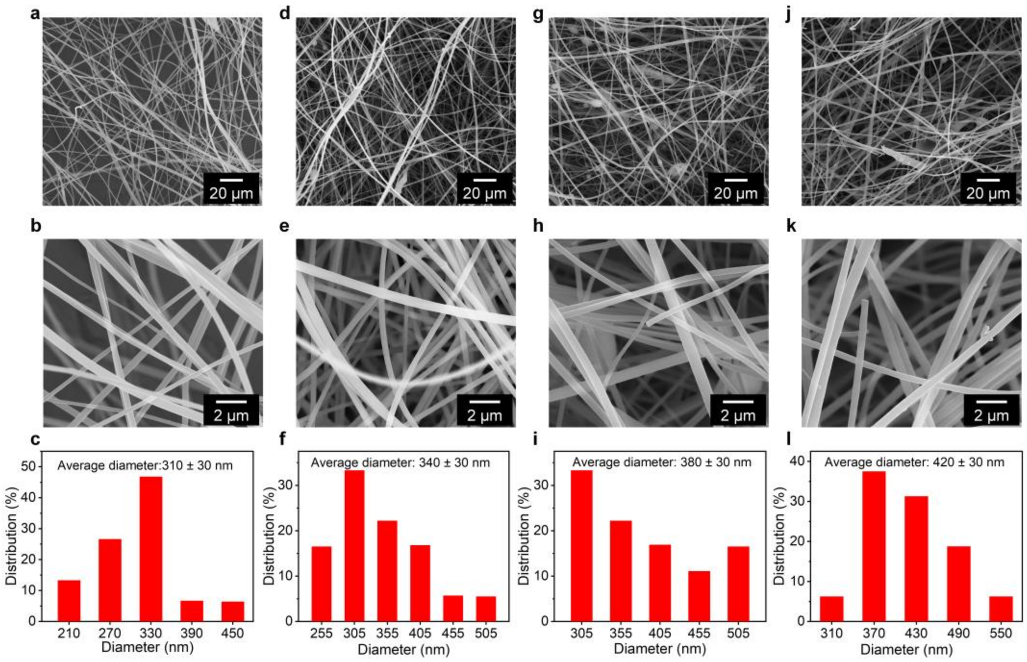

Supplementary Figure 5. Representative SEM images showing the surface micromorphology of mullite nanofibers with various injection speeds (a-b) 1 mL/h, (d-e) 1.5 mL/h, (g-h) 3 mL/h, and (j-k) 7 mL/h during electrospinning. The diameter distribution histogram of the injection speeds of (c) 1 mL/h, (f) 1.5 mL/h, (i) 3 mL/h, and (l) 7 mL/h revealed a typical increase in the average diameter of the nanofibers with higher injection speeds.

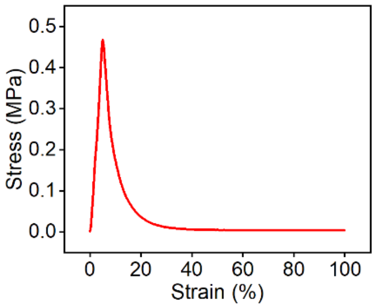

Supplementary Figure 6. Tensile mechanical properties of mullite nanofibrous membranes.

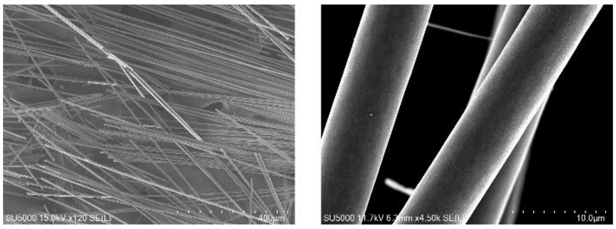

Supplementary Figure 7. SEM images showing the microscopic architectures of Al_2O_3 macro-fibers at different magnifications.

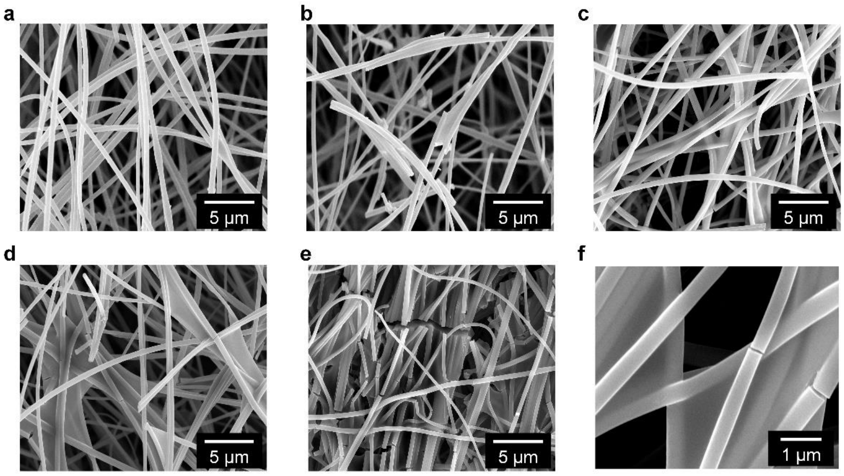

Supplementary Figure 8. The SEM images show the surface morphology of nanofibers under a concentration gradient of AlBSi sol (a) AlBSi- 0.5 wt%, (b) AlBSi-2 wt%, (c)AlBSi-4 wt%, (d) AlBSi-5 wt%, (e-f) AlBSi-8 wt%, revealing that AlBSi agglomerates between the fibers and becomes increasingly visible as the concentration increases from 0.5 wt% to 8 wt%, and some cracks appeared in samples prepared at 8 wt%.

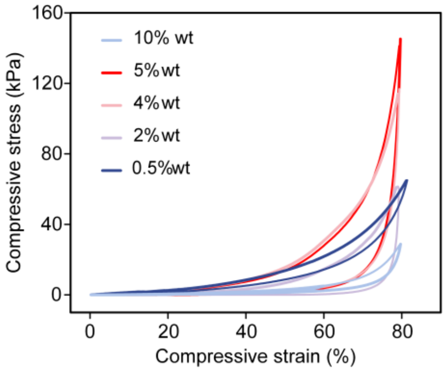

Supplementary Figure 9. Compressive stress-strain curves of BcF-CAs with AlBSi–0.5 wt%, AlBSi–2 wt%, AlBSi–4 wt%, AlBSi–5 wt%, AlBS–10 wt%.

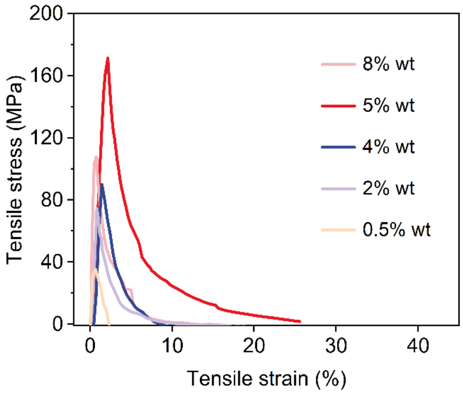

Supplementary Figure 10. Tensile stress-strain curves of BcF-CAs with (a) AlBSi–0.5 wt%, (b) AlBSi–2 wt%, (c) AlBSi–4 wt%, (d) AlBSi–5 wt%, (e) AlBS–8 wt%.

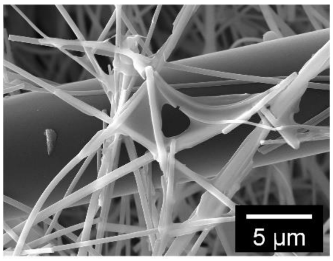

Supplementary Figure 11. The robust bonding between macro-fibers and nanofibers.

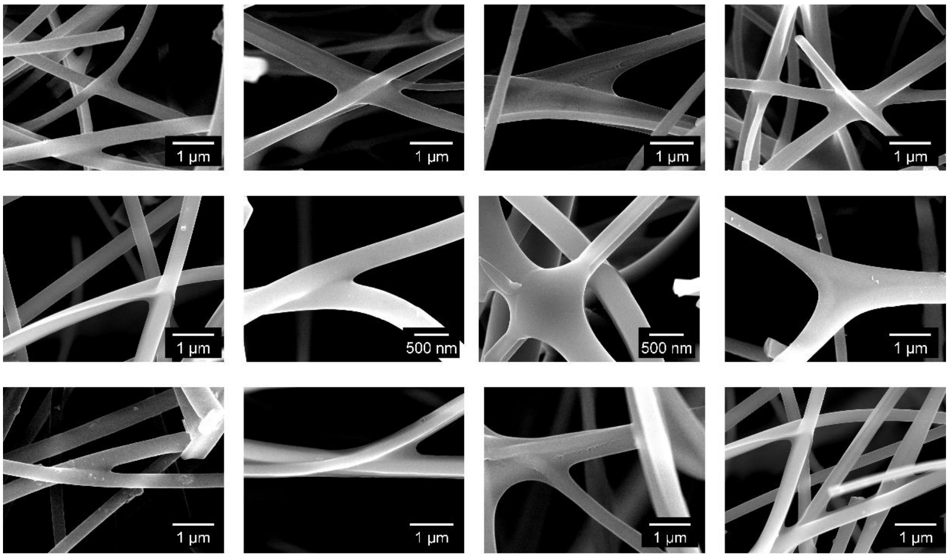

Supplementary Figure 12. FE-SEM images of several representative bonding points between the nanofibers of BcF-CAs.

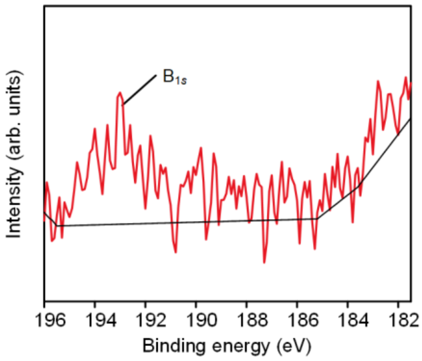

Supplementary Figure 13. XPS fine spectrum scan of B1s BcF-CAs with the binding energy ranging from 182 to 196 eV. The black line represents the Background baseline of the fine spectrum scan.

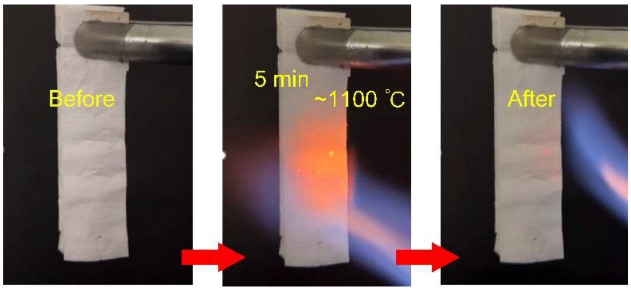

Supplementary Figure 14. The BcF-CAs were exposed to a butane blowtorch before and after the treatment.

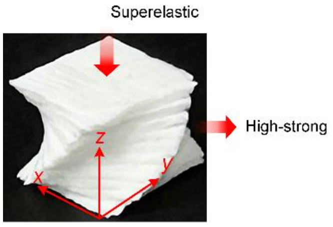

Supplementary Figure 15. The anisotropic mechanical properties of the BcF-CAs.

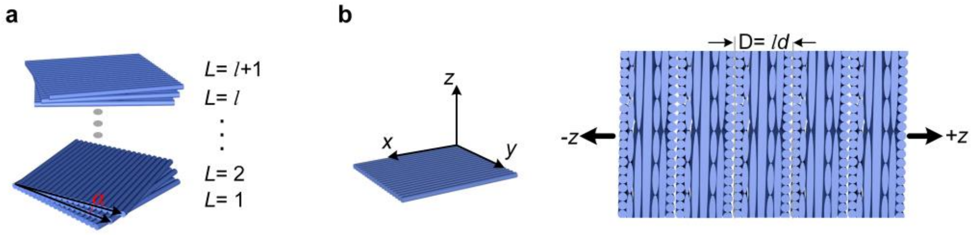

Supplementary Figure 16. (a) Diagram of the rotation for different layers showing that the fiber laminas of the chiral array started from L = 1 to L = l+1 (L is the number of fiber laminates), the helix of each L_{th} layer formed an angle ϕ= (L-1) α, and L layers were twisted by 180° (ϕ = 180° or an integer multiple of 180°) over a pitch distance D, where D = l d and d is the thickness of the layer. (b) Unit cell repetition along the z-axis for a = 30°, revealing that there were six cycles of the same rotation (l = 6) of the fiber array around the z-axis, indicating that the fiber laminas started from the x-axis with different rotations for the next six layers and returned to the x-axis for the seventh layer (L=7).

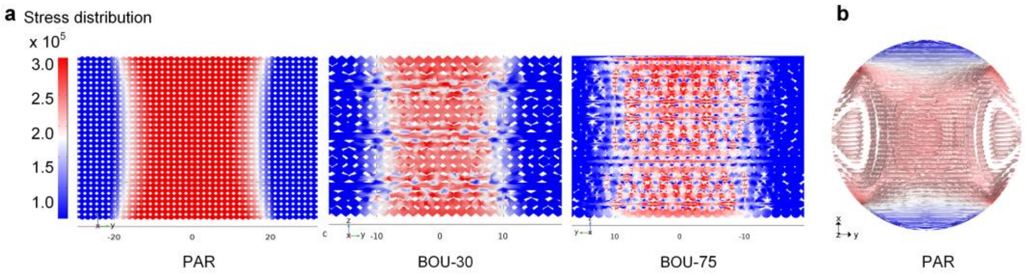

Supplementary Figure 17. (a) Stress nephograms of PAR, BOU-30 BOU-75 depicting the changes of axial stress in the *y*-*z* plane, showing that the maximum stress decreased from PAR to BOU-75. (b) The iso-surface map of the stress field showing the anisotropic tensile mechanical properties of the PAR array, i.e., the structure could withstand a high level of loading only when the direction of the force was aligned with the fiber orientation.

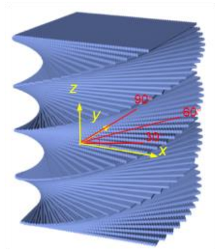

Supplementary Figure 18. The BOU-15 was subjected to tensile stress in different directions (30°, 60°, 90°), where red lines represent the directions of the force applied.

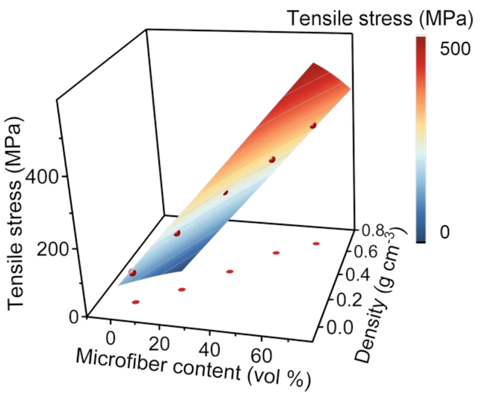

Supplementary Figure 19. 3D surface plots of the tensile stress as a function of *c* and density.

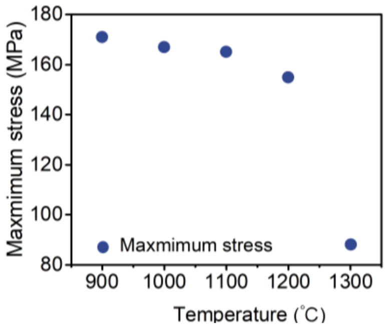

Supplementary Figure 20. Maximum stress of the BcF-CAs as a function of temperature.

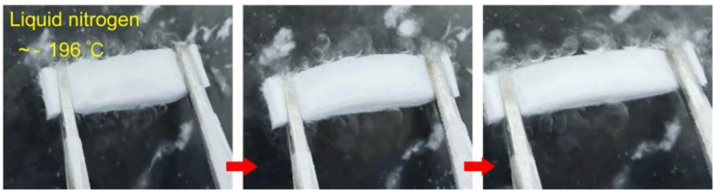

Supplementary Figure 21. Temperature-invariant flexibility. Bending and recovery processes in liquid nitrogen.

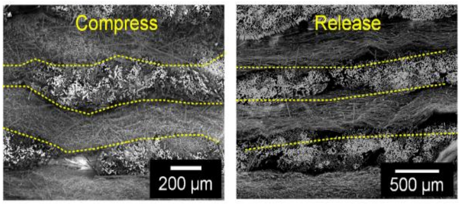

Supplementary Figure 22. The SEM images of arcuate-like hierarchical microstructure in BcF-CAs.

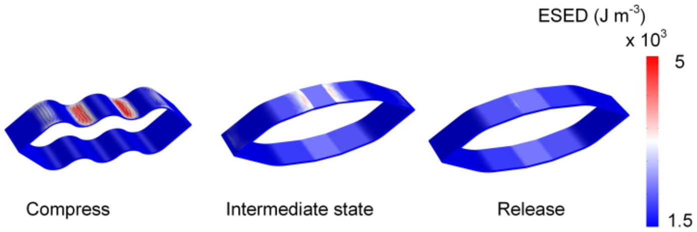

Supplementary Figure 23. The elastic strain energy density（ESED） profiles for the strain of 40% during the compression and release processes, respectively.

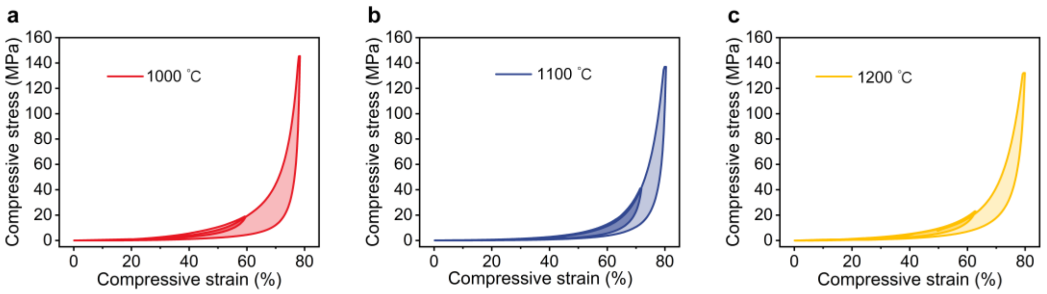

Supplementary Figure 24. The BcF-CAs were annealed at temperatures of (a) 1000 °C, (b) 1100 °C and (c) 1200 °C for 1 h, and the compressive loading-unloading behavior was evaluated uniaxially with a compression strain of 80%, showing that there was no significant difference over the entire temperature range (1000-1200 °C), which demonstrates their good thermal insulations.

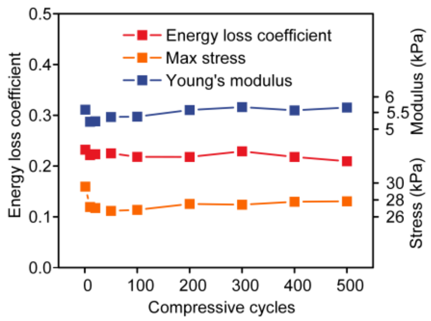

Supplementary Figure 25. Young's modulus, energy loss coefficient, and maximum stress as a function of compression cycles.

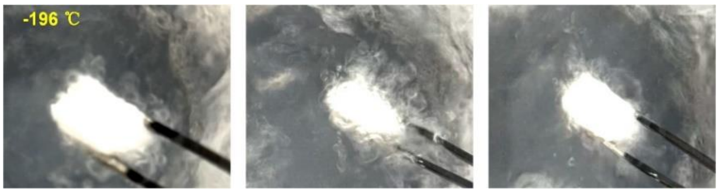

Supplementary Figure 26. Temperature-invariant flexibility. Bending and recovery processes in liquid nitrogen.

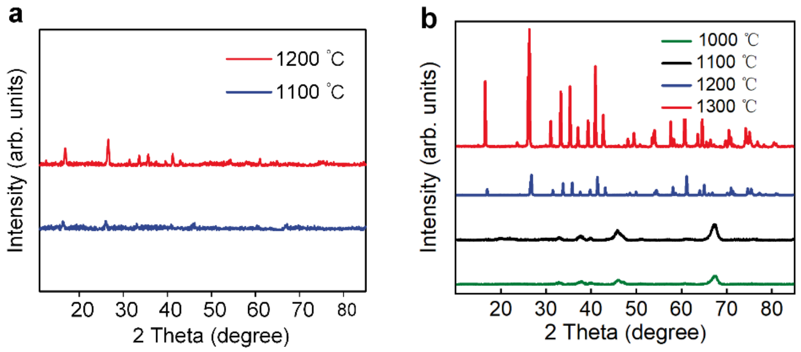

Supplementary Figure 27. (a) XRD spectra of mullite nanofibers at 1100-1200 °C for 1 h, showing that even after prolonged annealing at temperatures as high as 1200 °C, the characteristic peaks remained consistent with those of single mullite crystals, highlighting the superior thermal stability of mullite nanofibers under extreme conditions. (b) The XRD spectra of Al_2O_3 macrofibers at 900 °C to 1300 °C for 1 h, demonstrate that the Al_2O_3 macrofibers possess high thermal stability as nanofibers.

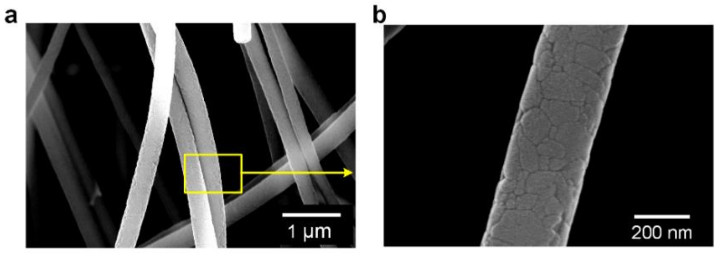

Supplementary Figure 28. SEM images of BcF-CAs after exposure to 1300 °C for 1 h at low magnification (a) and high magnification (b), showing that after calcining BcF-CAs at 1300 °C, oversized crystalline grains were formed, which resulted in some brittle fracture in the mullite nanofibers.

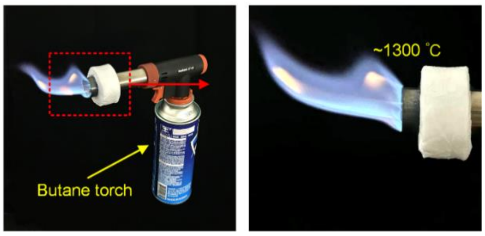

Supplementary Figure 29. Photograph of the setup for the measurement of thermal insulation performance of BcF-CAs.

Supplementary Table

Supplementary Table 1. The relevant properties of BcF-CAs and other insulation aerogels materials.

Materials	Maximum working temperature (℃)	Volume density (g/cm^3)	Tensile strength (MPa)	Thermal conductivity ($Wm^{-1}K^{-1}$)
BcF-CAs	1200	0.211-0.283	98-213	0.037
SiO_2 nanofibrous aerogels by freeze-shaping	1100	0.012-10	0.00417	0.023-0.032
ZrO_2-SiO_2 nanofibrous aerogels by immerse-stacking	1100	0.026	0.34	0.024
Binary-network structured SiO_2 nanofibrous aerogels (BSA)	1100	0.001	0.00214	0. 2196-0.28
Multifunctional core-shell nanorod aerogels	1200	0.128	1.6	0.024
SiC nanowire aerogel	900-1200	0.005-0.057	0.017-0.09	0.026
Hypocrystalline zircon nanofibrous aerogels（ZAGs）	1300	0.026	0.0424	0.026
Mullite nanofibrous aerogels via 3D reaction electrospinning（ICCAs）	1400	0.022-0.027	0.0127	0.0228
Porous carbon material	350	0.011	0.00045	/
Carbon aerogels	800	0.0057	0.007	/
